# Clinicopathological characteristics of surgically treated localized renal masses in patients previously exposed to chemotherapy

**DOI:** 10.1590/S1677-5538.IBJU.2018.0126

**Published:** 2019-04-01

**Authors:** Efrat Tsivian, Matvey Tsivian, Christina Sze, Ariel Schulman, Thomas J. Polascik

**Affiliations:** 1 Division of Urology, Department of Surgery, Duke University Medical Center, Durham, USA

**Keywords:** Carcinoma, Renal Cell, Chemotherapy, Cancer, Regional Perfusion, Kidney Neoplasms

## Abstract

**Purpose:**

To explore the potential association between renal mass characteristics and a history of chemotherapy.

**Materials and methods:**

A retrospective review of records of patients surgically treated for a localized renal mass between 2000 and 2012 was undertaken following an institutional review board approval. Patients age and sex, renal mass clinical characteristics (radiological size and mode of presentation) and pathological characteristics (diagnosis, renal cell carcinoma subtype, Fuhrman grade and stage) were compared between patients with and without a history of chemotherapy, using Fisher's exact test, Student's t-test and Wilcoxon rank sum test. A multivariate logistic analysis was performed to evaluate the independent association of chemotherapy and tumor pathology.

**Results:**

Of the 1,038 eligible patients, 33 (3%) had a history of chemotherapy. The distribution of clinical stage, renal mass diagnosis, renal cell carcinoma subtype, Fuhrman grade, pathological stage, sex and median age were similar between the general population and the chemotherapy group. However, the latter had a higher rate of incidental presentation (P = 0.003) and a significantly smaller median radiological tumor size (P = 0.01). In a subset analysis of T1a renal cell carcinoma, the chemotherapy group presented an increased rate of high Fuhrman grade (P = 0.03). On multivariate analysis adjusted for radiological tumor size, sex and age the chemotherapy cohort had a 3.92 higher odds for high Fuhrman grade.

**Conclusion:**

Patients with a history of chemotherapy typically present with smaller renal masses that, if malignant, have higher odds of harboring a high Fuhrman grade and thus may not be suitable for active surveillance.

## INTRODUCTION

In the US, kidney cancer affects approximately 63,990 new patients each year and causes the death of more than 14,000 on an annual basis (1). In the general population, the most frequent malignant histological subtype is the clear cell renal cell carcinoma followed by the papillary and chromophobe subtypes (2). However, the clinicopathological characteristics of renal masses and specifically of renal cell carcinoma have been shown to be different in specific subpopulations (3–5).

A positive medical history of chemotherapy has been associated with an increased risk of developing secondary malignancies including kidney cancer (6–9). Still, the clinicopathological characteristics of renal masses in patients with a history of chemotherapy have yet to be described. Chemotherapeutic agents may affect the clinicopathological profile of renal masses through several mechanisms. The nephrotoxic effect that some chemotherapeutic agents have in the presence of additional insulting agents could lead to chronic renal damage (10, 11) that, can be associated with a higher prevalence of papillary renal cell carcinoma and better oncological outcomes (5). On the other hand, renal masses in patients with past chemotherapy exposure can also present a completely distinct and possibly a more aggressive clinicopathological profile due to the carcinogenic effect some chemotherapeutic agents may exert on renal cells (12). While most evidence on chemotherapeutic carcinogenicity is associated with hematologic malignancies, specific chromosomal translocations have been documented in renal masses in pediatric patients following chemotherapy (13).

In this study, we aim to describe the clinical and pathological characteristics of renal masses in individuals with a history of chemotherapy and compare them to those of the general population in order to assess if they require any special considerations when deciding on their management.

## MATERIALS AND METHODS

### Cohort

Following approval from the institutional review board, records of patients who underwent extirpative therapy for a localized renal mass at our institution between 2000 and 2012 were reviewed. Patients included in the study were those who underwent partial or radical nephrectomy for a renal mass. Patients treated with ablation, who had a hereditary syndrome such as Von Hippel - Lindau, or had a locally advanced mass were excluded from the study. The following variables were collected: age, sex, history of chemotherapy, mode of presentation (incidental vs. symptomatic), clinical stage, radiological size, pathological diagnosis (malignant vs. benign), renal cell carcinoma (RCC) subtype, Fuhrman grade and pathological stage.

### Statistical analysis

Patient and renal mass characteristics were compared between patients with and without history of chemotherapy using Chi-square and Fisher's exact test for categorical data as well as Student's t-test and Wilcoxon rank sum test for continuous data as appropriate. In addition, a subgroup analysis compared patient and disease characteristics in a cohort of patients with small renal masses (SRM). The data are reported as median (interquartile range) or number (%). Finally, a multivariate logistic analysis was done in order to assess the association of medical history of chemotherapy and Fuhrman grade while accounting for confounding parameters (i.e. sex, age, malignant mass (RCC) radiological size). Fuhrman grade was categorized as low (1–2) and high (3–4). All tests were 2-tailed. P < 0.05 was considered statistically significant. Analyses were performed using the R v3.3.1 software (the R Foundation for Statistical Computing, Vienna, Austria) using “Hmisc” and “gmodels” libraries.

## RESULTS

Of the 1,652 available records, 1.038 met the inclusion criteria and were reviewed. The cohort was predominantly male (59%) with a median age of 61 years. Only 33 (3%) patients of the total cohort had a positive medical history for chemotherapy. Patient and renal mass characteristics are detailed in Table-1. There was no significant difference in the distribution of sex (p = 0.4), clinical and pathological stage (p = 0.4 and 0.5 respectively), renal mass pathological diagnosis (p = 1), RCC subtype (p = 1) and Fuhrman grade (p = 0.09). However, renal masses in patients with a medical history of chemotherapy were more frequently diagnosed incidentally (97% vs. 77%, p = 0.003) and demonstrated a significantly smaller median radiological size (3.1 vs. 4cm, p = 0.01).

**Table 1:** Patient and renal mass characteristics in the total cohort.

Variable		Total	Chemo+	Chemo-	P value
Number of patients		1038	33 (3%)	1005 (97%)	
**Gender**					**0.4**
	Male	617 (59%)	17 (52%)	600 (60%)	
	Female	421 (41%)	16 (48%)	405 (40%)	
Median Age (IQR)		61 (52-68)	63 (56-66)	60 (52-68)	0.4
**Mode of presentation**					**0.003**
	Incidental*	801 (77%)	32 (97%)	769 (77%)	
	Symptomatic	237 (23%)	1 (3%)	236 (23%)	
Median radiological size in cm		4 (2.7-6.5)	3.1 (1.8-4.9)	4 (2.7-6.6)	0.01
**Clinical Stage**					**0.4**
	T1a	540 (52%)	20 (61%)	520 (52%)	
	T1b	275 (26%)	10 (30%)	265 (26%)	
	T2a	120 (12%)	2 (6%)	118 (12%)	
	T2b	103 (10%)	1 (3%)	102 (10%)	
**Renal mass diagnosis**					**1**
	Benign	187 (18%)	6 (18%)	181 (18%)	
	Malignant	851 (82%)	27 (82%)	824 (82%)	
**RCC subtype**					**1**
	Clear Cell	631 (74%)	22 (81%)	609 (74%)	
	Papillary	180 (21%)	5 (19%)	175 (21%)	
	Chromophobe	23 (3%)	0 (0%)	23 (3%)	
	Other	17 (2%)	0 (0%)	17 (2%)	
**Fuhrman grade**					**0.09**
	1	166 (20%)	3 (11%)	163 (20%)	
	2	492 (58%)	14 (52%)	478 (58%)	
	3	151 (18%)	6 (22%)	145 (18%)	
	4	42 (5%)	4 (15%)	38 (5%)	
**Pathological stage**					**0.5**
	T1a	406 (48%)	13 (48%)	393 (48%)	
	T1b	189 (22%)	9 (33%)	180 (22%)	
	T2a	59 (7%)	0 (0%)	59 (7%)	
	T2b	39 (5%)	1 (4%)	38 (5%)	
	≥ T3	158 (19%)	4 (15%)	154 (19%)	

* Incidental presentation includes patients that were found to have a renal mass during follow-up visits (3 of those with positive history of chemotherapy).

**Figure 1:**
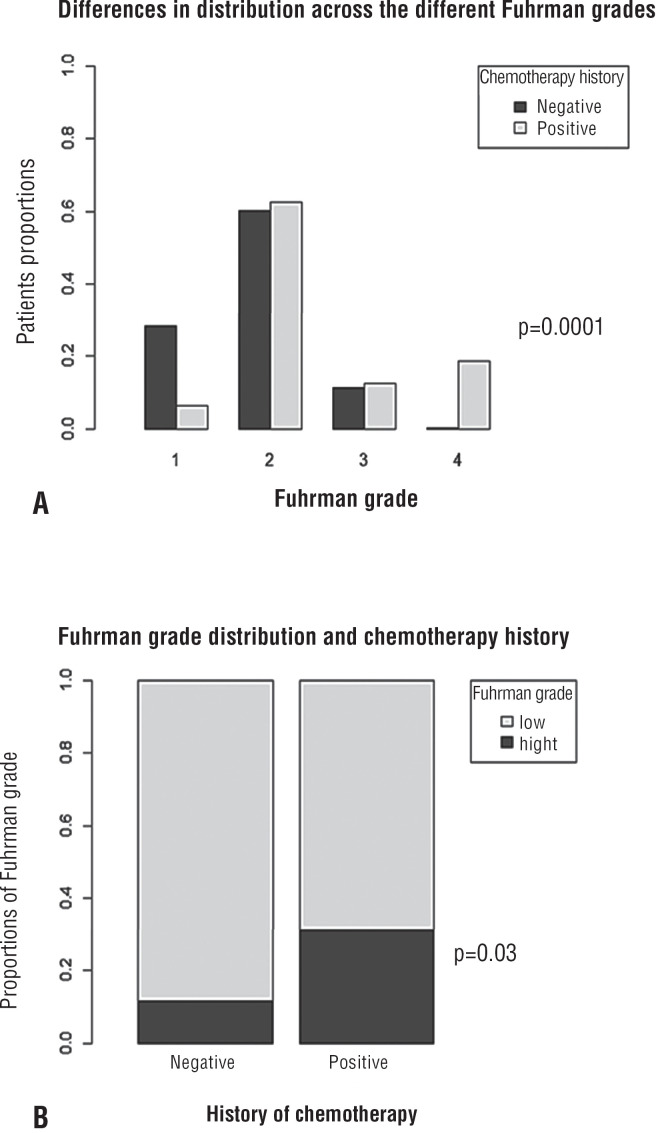
The distribution of Fuhrman grade was significantly different between the chemotherapy group and the general population (1a). The chemotherapy group had significantly higher rates of high Fuhrman grade (1b).

When renal mass and patient characteristics were compared in the subgroup of patients with SRM, median radiological size remained significantly different between those who underwent chemotherapy in the past and those who did not (2 vs. 2.8cm, p = 0.009) (Table-2). On the other hand, the distribution of the mode of presentation was similar between the two patient groups as well as the median age and the distribution of sex, renal mass diagnosis, RCC subtype and pathological stage (p = 0.3, 0.9, 0.6, 1, 0.9, 0.2 respectively). Interestingly, in the SRM subgroup, Fuhrman grade distribution was revealed to be different with higher rates of high Fuhrman grade in the chemotherapy group (31% vs. 12%, p = 0.03) (Figures 1a-b). In the multivariate analysis that aimed to evaluate whether a history of chemotherapy remains significantly associated with Fuhrman grade when adjusting for known confounders (i.e sex, age, renal mass radiological size), patients with RCC and a history of chemotherapy had 3.92 (CI 1.16-11.71, p = 0.02) higher odds of harboring a high Fuhrman grade (Table-3).

**Table 2:** Patient and renal mass characteristics in the SRM cohort.

Variable		Total	Chemo+	Chemo-	P value
Number of patients	540	20 (4%)	520 (96%)	
**Gender**					**0.6**
	Male	248 (59%)	8 (50%)	240 (59%)	
	Female	175 (41%)	8 (50%)	167 (41%)	
	Median Age (IQR)	60 (51-68)	62 (54.5-65.3)	59.5 (51-68)	0.9
**Mode of presentation**					**0.3**
	Incidental*	483 (89%)	20 (100%)	463 (89%)	
	Symptomatic	57 (11%)	0 (0%)	57 (11%)	
Median radiological size in all SRM in cm (IQR)		2.7 (2-3.2)	2 (1.7-2.7)	2.8 (2-3.3)	0.009
Median radiological size in malignant SRM in cm (IQR)		2.8 (2-3.3)	1.8 (1.7-2.7)	2.8 (2.1-3.3)	0.006
**Renal mass diagnosis**					**1**
	Benign	117 (22%)	4 (20%)	113 (22%)	
	Malignant	423 (78%)	16 (80%)	407 (78%)	
**RCC subtype**					**0.9**
	Clear Cell	305 (72%)	13 (81%)	292 (72%)	
	Papillary	100 (24%)	3 (19%)	97 (24%)	
	Chromophobe	10 (2%)	0 (0%)	10 (2%)	
	Other	8 (2%)	0 (0%)	8 (2%)	
**Fuhrman grade**					**0.0001**
	1	116 (27%)	1 (6%)	115 (28%)	
	2	255 (60%)	10 (62%)	245 (60%)	
	3	48 (11%)	2 (12%)	46 (11%)	
	4	4 (1%)	3 (19%)	1 (0%)	
**Dichotomized Fuhrman grade:**					
	Low (1-2)	371 (88%)	11 (69%)	360 (88%)	0.03
	High (3-4)	52 (12%)	5 (31%)	47 (12%)	
**Pathological stage**					**0.2**
	T1a	372 (88%)	12 (75%)	360 (88%)	
	T1b	21 (5%)	2 (12%)	19 (5%)	
	T2a	2 (0%)	0 (0%)	2 (0%)	
	T2b	0 (0%)	0 (0%)	0 (0%)	
	≥ T3	28 (7%)	2 (12%)	26 (6%)	

* Incidental presentation includes patients that were found to have a renal mass during follow-up visits (3 of those with positive history of chemotherapy).

**Table 3:** Multivariable analyses to evaluate the association of Fuhrman grade and a history of chemotherapy in localized RCC, adjusted for age, sex and radiological size.

Factor		OR (95% CI)	P value
History of chemotherapy			0.02
	No	Reference	
	Yes	3.92 (1.16-11.71)	
Radiological size in cm		1.25 (0.85-1.85)	0.2
**Sex**			0.1
	Female	Reference	
	Male	1.11 (0.61-2.04)	
Age		1.02 (1.00-1.05)	0.1

## DISCUSSION

Fung et al. and van den Beit-Dusebout et al. described an increased risk for the development of secondary malignancies, in particular hematologic malignancies after chemotherapy with a median latency of 12.5 and 17.6 years respectively (7, 9). It has also been reported that chemotherapy increases the risk of renal cancer in childhood cancer survivors (7, 14). The effect of chemotherapy on the clinicopathological characteristics of renal masses has yet to be reported. In this study, we aimed to evaluate whether patients with a previous exposure to chemotherapy may present with a renal mass that is clinically and pathologically different from those in the general population.

This study's cohort included only 33 (3%) patients with a history of chemotherapy surgically treated for a localized renal mass at our institution. While most of the patients and renal mass characteristics that the chemotherapy group presented did not differ from those of the general population, some peculiarities were noticed. Renal masses in patients who were exposed to chemotherapy in the past were more frequently diagnosed incidentally. Moreover, their median radiological size was significantly smaller. These findings are not surprising since patients with a history of chemotherapy are under surveillance for their primary malignancy and thus, undergo imaging tests more frequently than the general population. In fact, the rising incidence of renal masses is considered to be partly due to the increase in cross sectional imaging (15, 16).

Interestingly, when renal mass and patient characteristics were compared in only the SRM cohort it was noticed that patients with a history of chemotherapy still presented with significantly smaller masses. Furthermore, when only malignant SRMs were examined, these were characterized by a higher rate of high Fuhrman grade, which is known to be independently associated with RCC biological behavior (17, 18). Current literature is lacking in evidence that could explain the study's findings. However, studies that explored the development of secondary malignancies following chemotherapy report an increased risk of kidney cancer following platinum-based therapy (7). Renal cells may be affected by the toxicity of platinum-based chemotherapies due to their exposure. In fact, the kidney is the primary means for short and long-term cisplatin excretion (19). In addition, studies have described the persistence of partially reactive circulating platinum even after 10 years following completion of chemotherapy (20, 21) and have documented the presence of platinum-DNA adducts in different human tissues including the kidney (21, 22) that could contribute to the different pathological profile that RCC demonstrates in patients with a history of chemotherapy. In this study, platinum-based chemotherapeutic agents were used in about a third of the chemotherapy cohort patients (Table-4). Other chemotherapeutics that these patients were exposed to included alkylating agents (Lomustine and Cyclophosphamide) and topoisomerase II inhibitors (Etoposide) that are known to contribute to the development of secondary malignancies (23, 24). However, none of these chemotherapeutics was shown to be associated with the pathological characteristics of renal masses.

**Table 4:** Primary malignancies and chemotherapy characteristics.

		Rate (Percentage) / Median(IQR)
Primary Malignancy:		
Anal squamous cell carcinoma		1 (3%)
Lymphoma*		5 (15%)
Breast		10 (30%)
Cervical carcinoma		1 (3%)
Cholangiocarcinoma		1 (3%)
Colon		3 (9%)
Esophageal carcinoma		1 (3%)
Fallopian tube carcinoma		1 (3%)
Glioblastoma		1 (3%)
Lung carcinoid		1 (3%)
Lung carcinoma		4 (12%)
Tongue squamous cell carcinoma		1 (3%)
TCC		2 (6%)
Unknown origin		1 (3%)
Combined chemotherapy with radiotherapy		7 (21%)
Median number of years from chemotherapy to surgery		3 (2-11)
**Platinum based chemotherapy**		
	Yes	12 (36.4%)
	No	14 (42.4%)
Unknown		7 (21.2%)
**Total**		**33**

* Lymphoma includes: Hodgkin and non-Hodgkin lymphoma

There are several limitations to this study that need to be acknowledged. First, selection bias may be present due to the retrospective nature of the study. In addition, the study is based on a cohort treated at a referral center and thus, this study's findings may not be extrapolated to other populations. Extrapolation of the findings to the general population may also be difficult due to the limited number of patients with a history of chemotherapy used in the analysis. Since the chemotherapy group only included 33 patients with different primary malignancies and treatment plans, confounding factors such as the type of chemotherapeutic agents, dosage used, and time from chemotherapy to renal mass diagnosis could not be accounted for. Also, due to the cohort size there was an insufficient power to evaluate how the study's findings correlate to the oncological control of malignant SRMs in patients with a history or chemotherapy. Despite these limitations, our study provides initial evidence on the possible association between medical history of chemotherapy and the biological characteristics of RCC in the context of SRM. Clinically, the study's findings may indicate that patients with a history of chemotherapy may not be the ideal candidates for active surveillance since they have higher odds for a disease that is histologically more aggressive. Further studies are necessary in order to clarify the impact of past exposure to chemotherapy on the survival of patients managed with active surveillance for their SRM.

## CONCLUSIONS

In this study, a history of chemotherapy was associated with renal masses that were more frequently incidental and of smaller radiological size. In addition, in the SRM subset chemotherapy was significantly associated with high Fuhrman grade. Additional studies are necessary in order to clarify the biological mechanisms through which chemotherapy may contribute to the more aggressive profile of T1a RCC. Furthermore, future studies are required in order to examine how chemotherapy may have a role in survival outcomes of patients with localized RCC.

## ABBREVIATIONS

RCC: = Renal Cell Carcinoma
SRM: = Small Renal Mass
